# SB772077B (SB77) Alleviated the Aqueous Outflow Resistance Mediated by Cyclic Mechanical Stress in Perfused Human Cadaveric Eyes

**DOI:** 10.1038/s41598-020-67087-2

**Published:** 2020-06-23

**Authors:** Soundararajan Ashwinbalaji, Ravinarayanan Haribalaganesh, Subbaiah Krishnadas, Veerappan Muthukkaruppan, Srinivasan Senthilkumari

**Affiliations:** 1 Department of Ocular Pharmacology, Aravind Medical Research Foundation, #1, Anna Nagar, Madurai-20, Tamilnadu, India; 2 Glaucoma Clinic, Aravind Eye Hospital, #1, Anna Nagar, Madurai-20, Tamilnadu, India; 3 Advisor, Aravind Medical Research Foundation, #1, Anna Nagar, Madurai-20, Tamilnadu, India

**Keywords:** Biotechnology, Molecular biology, Medical research

## Abstract

The intraocular pressure lowering property of a new rho kinase inhibitor, SB772077B (SB77) has been previously demonstrated in perfused human cadaveric eyes. In this study, the efficacy of SB77 in alleviating the aqueous outflow resistance mediated by cyclic mechanical stress in perfused human cadaveric eyes was investigated. A human anterior segment perfusion culture model was used to investigate the effect of cyclic intraocular pressure (IOP) on aqueous outflow facility in presence or absence of SB77. The status of RhoA activation and the downstream effector molecule myosin-light chain phosphorylation (p-MLC) was investigated by Western blot. Cyclic mechanical stress resulted in decrease in aqueous outflow facility (–19.79 ± 4.93%; *p* = 0.019) in perfused human eyes and treatment with SB77 (50 µM) significantly enhanced outflow facility by 15% (p = 0.05). The increase in outflow facility by SB77 was confirmed with the inactivation of RhoA/ROCK signaling and decreased expression of extracellular matrix markers. SB77 effectively reduced the outflow resistance mediated by cyclic IOP and thus may be a potential clinical candidate for the management of glaucoma.

## Introduction

Intraocular pressure (IOP) is the only modifiable risk factor for the management of glaucoma and all current treatment strategies are aimed to lower IOP. In the eye, the IOP is generated and maintained as a balance between the aqueous humor formation and drainage through the outflow pathways (conventional outflow pathway and uveoscleral outflow pathway). The trabecular meshwork (TM) at the irido-corneal angle forms the conventional outflow pathway which is the primary site for the aqueous humor to exit from the anterior chamber of the eye^1–3^. The TM outflow pathway provides resistance to the aqueous humor outflow and the majority of the resistance resides in the inner wall region of TM, which comprises the juxtacanalicular connective tissue (JCT) and the inner wall endothelium of Schlemm’s canal (SC)^4^.

Several studies support the concept that aqueous outflow is pulsatile in nature and corresponds to transient oscillatory or cyclic IOP fluctuations such as those that occur with the ocular pulse, blinking and eye movements^5,6^. Pulsatile aqueous flow mechanisms are observable and well documented *in vivo* in both normal and glaucomatous eyes^6^. The pulsatile outflow is reduced in glaucomatous condition which is reported to be restored by some glaucoma medications such as miotics, adrenergic and prostaglandins. The restoration of pulsatile outflow is followed by the reduction of IOP^6^. In addition, a significant alterations in the composition of extracellular matrix (ECM) and TM cellularity contributes to changes in TM tissue level biomechanical properties which indirectly affect the pulsatile outflow^7^. The mechanism by which the TM cells sense and respond to cyclical biomechanical stress is not clearly understood.

Experimentally induced cyclic IOP altered or decreased the conventional aqueous outflow in an *ex vivo* model of human and porcine anterior segment organ cultures^8^. The mechanism by which the cyclic mechanical stress affects the outflow facility is not completely understood. However, it is proposed that the decreased outflow facility during stress condition is due to the abnormal accumulation of extracellular matrix (ECM) which resulted in TM stiffness. A significant positive correlation between the outflow resistance and TM stiffness in humans and mice were observed^9,10^. Cyclic stress also affected TM cell contractility in cultured TM cells^11^. Laboratory studies are expected to provide a meaningful insight into how the cellular responses are regulated in response to cyclic mechanical stress. Modulation of such responses may pave the way for restoring the IOP homeostasis in glaucoma.

RhoA is a member of the Rho family of 20 to 30 kDa GTPase proteins that switch between an active GTP-bound form and an inactive GDP-bound form. It is an important regulator of actin stress fibres^12^. The Rho-associated coiled-coil forming protein kinase (ROCK) is a downstream effector molecule of RhoA acts as a serine/threonine kinase and phosphorylates various substrates. Actin stress fibre reorganization is mediated by the ROCK pathway^13^. ROCK is involved in the synthesis of ECM components in the TM and permeability of Schlemm’s canal endothelial cells^14^. Activation of the RhoA/ ROCK pathway decreases aqueous humor outflow through the TM by inducing alterations in cell contraction, actomyosin assembly, cell adhesion and ECM synthesis. Primary molecules that transmit RhoA/ROCK signaling (e.g.: myosin light chain phosphatase, LIM kinase, cofilin) are expressed in human TM with mediators for this signaling pathway present in the aqueous humour^15–17^. Therefore inhibition of this pathway is an attractive strategy to increase outflow facility in the TM and reduce IOP in the management of glaucoma.

The involvement of RhoA/ROCK signaling in the pathogenesis of glaucoma is well established and led to the discovery of FDA approved Rho kinase inhibitors (RKI) such as ripasudil and netarsudil for glaucoma therapy^18,19^. In our previous study, the IOP lowering property of a new amino-furan Rho Kinase inhibitor SB772077B (SB77) was demonstrated in perfused human eyes under steady state perfusion^20^. The present study was aimed to investigate whether the SB77 alleviates the aqueous outflow resistance mediated by the cyclic mechanical stress. We hypothesize that the cyclic biomechanical stress (a physiologically relevant stress) increases outflow resistance by activating the RhoA/ROCK signaling and the treatment with SB77 reduce such resistance by inactivating the RhoA/ROCK signaling thus enhancing outflow facility in the TM. Interestingly, our results clearly indicate that experimentally induced cyclic stress reduced the aqueous outflow facility in perfused human cadaveric eyes. Treatment with SB77 significantly enhanced outflow facility which was substantiated by inactivation of RhoA/ROCK signaling and decreased expression of ECM markers. Thus SB77 may be a potential clinical candidate for the management of glaucoma.

## Results

The mean (±SD) age of the donors used for the study was 70.7 ± 14.5 years. The mean elapsed time between enucleation and culture was 26.3 ± 2.5 h except for 2 pairs of eyes (S. Table 1).

**Table 1:** Summary of Results from HOCAS in response to cyclic pulsation in the presence or absence of SB77.

Experimental Setup 1 - Cyclic Pulsation and Steady state flow comparison											
Donor Code	Age	Sex	Stable Baseline Before Pulse (µl/minute/mm Hg)		Pulse Duration (h)	Outflow Facility (24 h) (Normalized to Baseline)		OFR (EXP/CTL)		% Change in OF	
			Control (no pulse)	Cyclic pulse		Control (no pulse)	Cyclic pulse				
1	75	M	0.17	0.25	8	0.94	0.88	0.95		−5.44	
2	40	M	0.21	0.28	8	1.07	0.64	0.6		−39.92	
3	60	M	0.08	0.19	8	0.95	0.84	0.88		−11.62	
4	85	F	0.14	0.26	8	0.8	0.68	0.85		−14.92	
5	72	M	0.08	0.16	8	0.77	0.58	0.76		−24.23	
6	53	F	0.29	0.2	8	0.85	0.65	0.77		−22.66	
						**0**.**89** ± **0**.**04**	**0**.**71** ± **0**.**05**			−**19**.**8** ± **4**.**9**	
**Experimental Setup 2 - Cyclic Pulsation in Presence or Absence of SB77**											
**Donor Code**	**Age**	**Sex**	**Stable Baseline Before Pulse (µl/minute/mm Hg)**		**Pulse Duration (h)**	**Outflow Facility (24 h) (Normalized to Baseline)**			**% Change in OF**		
			**Cyclic pulse**	**Cyclic pulse** + **SB77**		**Cyclic pulse**	**Cyclic pulse** + **SB77**		**Cyclic pulse**		**Cyclic pulse** + **SB77**
1	72	M	0.15	0.19	8	0.85	1.27		−15.35		27.01
2	85	M	0.18	0.14	8	0.89	1.17		−10.53		16.67
3	70	M	0.09	0.14	8	0.91	1.11		−8.65		11.2
4	85	F	0.27	0.12	8	0.8	1.03		−19.95		2.82
5	81	M	0.08	0.08	8	0.74	1.19		−25.9		19.44
						**0**.**84** ± **0**.**06**	**1**.**15** ± **0**.**09**		−**16**.**1** ± **7**.**0**		**15**.**4** ± **9**.06*

Outflow facility (OF) (μl/minute/mm Hg) was calculated as the ratio between the inflow rate (μl/minute) and the measured IOP (mm Hg). OFR was calculated using the formula: Treated/BL/Control/BL. % Change in OF was calculated using the formula: [(Treated/BL/Control/BL) -1] X100.

An average decrease in outflow facility (−19.79 ± 4.93%; n = 6) was observed in response to IOP oscillations (experimental setup1). In another set of experiments, an overall decrease in outflow facility of −16 ± 7.0% (n = 5) was observed in anterior segments those received IOP pulsations. Treatment with 50 µM SB77 caused significant enhancement in outflow facility by 15% (p < 0.05) (experimental setup 2). Data are represented as Mean ± SEM; **p < 0.005; *p < 0.05; Paired-t-test. EXP – Experiment; CTL = Control; OFR – Outflow ratio.

### Cyclic IOP induces aqueous outflow resistance and the effect of sb77 in modulating the outflow resistance in perfused human cadaveric eyes

#### Outflow facility

**Figure 1:**
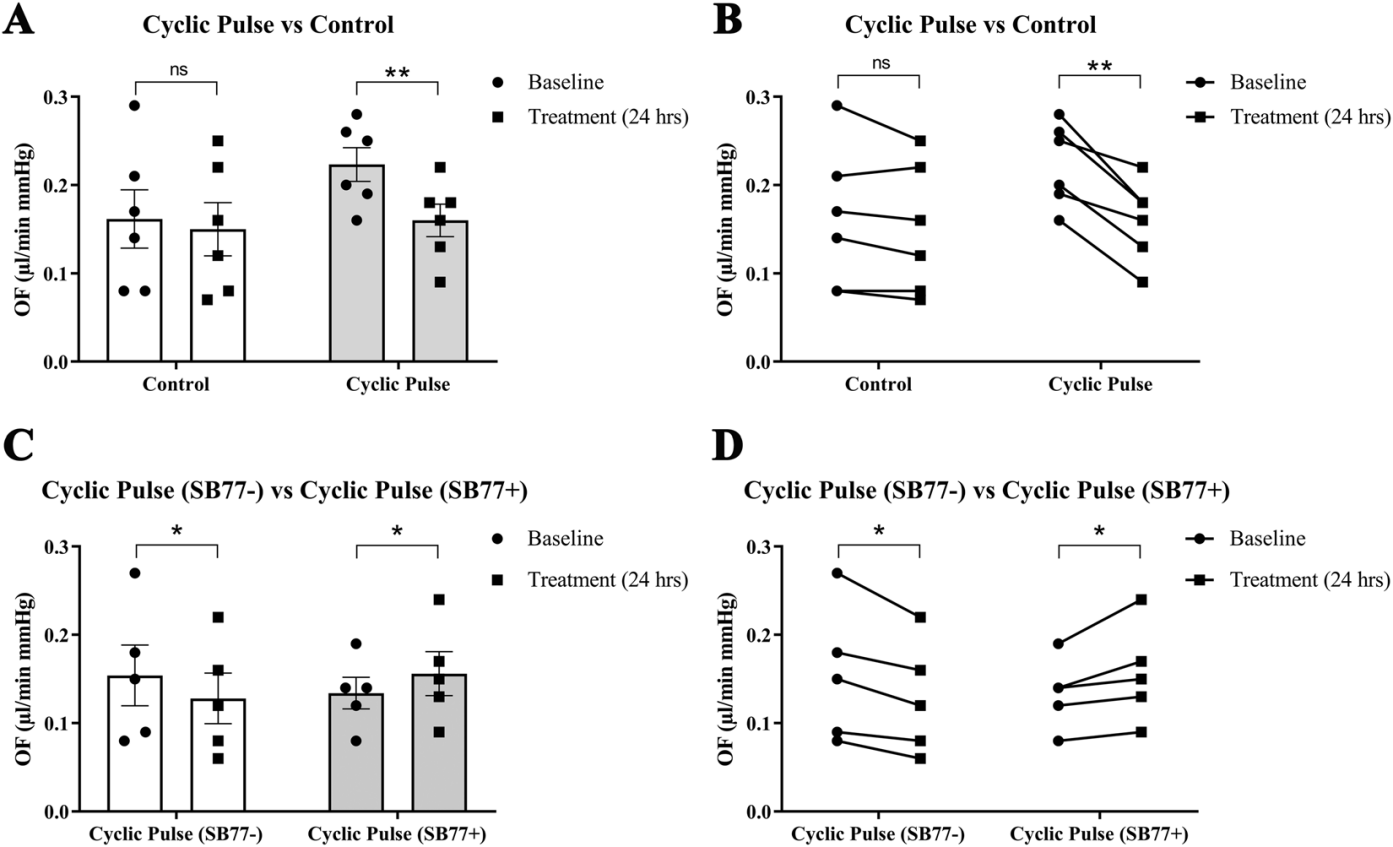
The effect of cyclic pulsations on aqueous outflow facility. The experimental anterior segment received IOP pulsations for 8 h. (**A**) The presence of experimentally induced cyclic mechanical stress significantly reduced the aqueous outflow facility as compared to its control (*p* = 0.003 (BL *vs* 24 hours); n = 6 pairs). Individual data points (black color dots) are indicated on the bar graph representing mean ± SEM. (**B**) Pairwise comparison between baseline and post cyclic pulsations for all treated pairs of eyes is represented. (**C**) Treatment with SB77 showed a significant increase in the outflow facility as compared to its control (*p* = 0.05 (BL *vs* 24 hours); n = 5 pairs). Individual data points (black color dots) are indicated on the bar graph representing mean ± SEM. **p < 0.005; *p < 0.05; Paired t-test. (**D**) Pairwise comparison of each pair of eyes received cyclic IOP pulsations in presence or absence of SB77 treatment is represented.

The effect of cyclic IOP on outflow facility was investigated in presence or absence of SB77 (50 µM). A representative IOP graph after cyclic IOP in presence or absence of SB77 is given in (Fig. S2). The mean outflow facility after cyclic pulse in experimental eyes showed a significant decrease in outflow facility (0.18 μl/minute/mm Hg) from its own baseline (0.24 μl/minute/mm Hg) (*p* = 0.003, which was monitored for 24 hours, whereas control eyes under steady state perfusion showed no significant difference in outflow facility (0.13 μl/minute/mm Hg) to its own baseline (0.15 μl/minute/mm Hg) (*p* = 1.191) (Fig. 1A). Change in outflow facility in experimental eyes were normalized to respective controls and expressed as ΔOFR (Experiment/Control). Human anterior segments showed an average decrease in percentage outflow facility (−19.79 ± 4.93%) (*p* = 0.019) in response to IOP oscillations as compared to control eyes under steady state perfusion (Experiment 1).

In another set of experiment, an overall decrease in outflow facility of −16 ± 7.0% (*p* = 0.019) was observed in anterior segments those received IOP pulsations. Treatment with 50 µM SB77 caused significant enhancement in outflow facility by 15% (*p* = 0.05); (Fig. 1B). A summary of the results of all human anterior segments subjected to cyclic IOP in presence or absence of SB77 is given in Table 1.

#### Tissue viability

**Figure 2:**
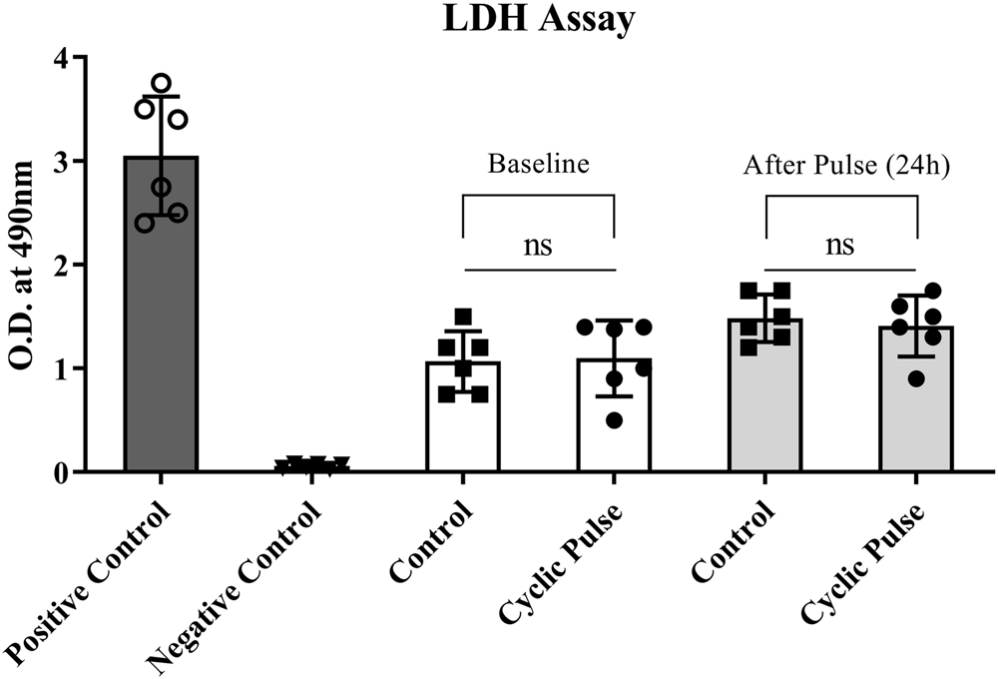
The effect of cyclic pulsations on tissue viability. The conditioned media collected at baseline and after cyclic IOP was assayed for LDH to check the tissue viability using commercially available kit as per the manufacturer’s instructions. There was a marginal increase in LDH activity before and after pulse in both experimental and control eyes. LDH activity was not statistically significant in eyes received cyclic IOP as compared to eyes on steady state perfusion showing no sign of toxicity induced by pulsation. Values are given as mean ± SD. ns, not statistically significant.

The effect of cyclic IOP on tissue viability was investigated using LDH assay. Our results indicate that the tissue viability was not affected by the cyclic IOP and found no significant difference in LDH activity between the experimental and control eyes (Fig. 2). This clearly indicates that the observed increase in outflow resistance is not because of the consequence of tissue damage but due to the active response of the outflow tissue to the cyclic mechanical stress.

### Cyclic IOP mediated alteration of RhoA/ROCK signaling and effect of SB77 in attenuating the stress response

**Figure 3:**
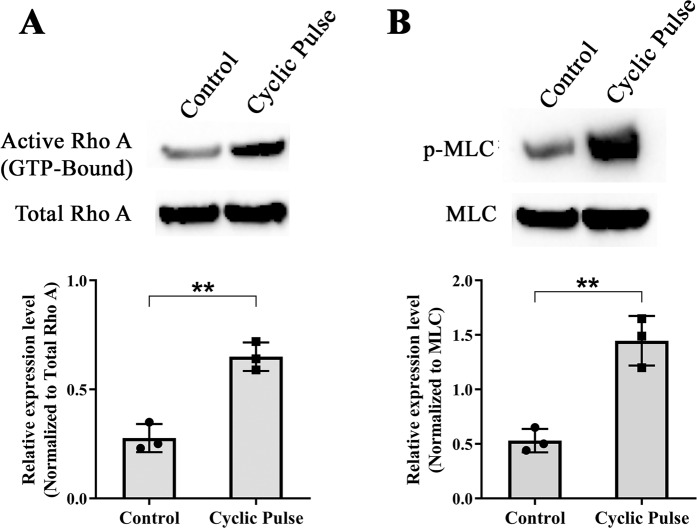
Effect of Cyclic Mechanical Stress on the Status of RhoA activation. Western blot analysis of (**A**) active RhoA, total RhoA and (**B**) RhoA downstream effector protein p-MLC. There was a significant increase in activated RhoA (*p* = 0.002) as well as p-MLC (*p* = 0.006) in TM tissue lysates obtained from the pulsed anterior segment as compared to its control. Levels of activated RhoA were normalized to total RhoA protein levels. p-MLC immunoblots were normalized to the total MLC loading control. The cropped images are used in the figure, and full length blots for the same are presented in S. Figure 5A,B. Individual data points (black color dots) are indicated on the bar graph representing mean ± SD. **p < 0.005; *p < 0.05; Student’s t-test; n = 3.

The effect of cyclic stress in the TM on RhoA/ROCK signaling was investigated by western blotting. Densitometry analysis showed a significant activation of RhoA (*p* = 0.002) as well as its downstream effector molecule p-MLC (*p* = 0.006) in the TM tissue lysates derived from the perfused human cadaveric eyes undergone cyclic IOP compared to eyes that received steady state flow (Fig. 3). This indicates that the experimentally induced cyclic IOP activated the RhoA/ROCK signaling which is further supported by the increase in outflow resistance.

**Figure 4:**
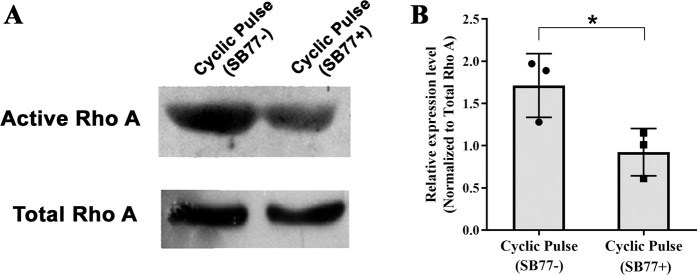
Effect of SB77 on the status of RhoA activation. (**A**)Western blot analysis of active RhoA and total RhoA in response to IOP pulsations with or without SB77 treatment. (**B**) Densitometry analysis showing the fold change in the expression of active RhoA. A significant decrease in active RhoA protein was observed in anterior segments received IOP oscillations in presence of SB77 as compared to its untreated eyes (*p* = 0.043). Levels of activated RhoA were normalized to total RhoA protein levels. The cropped images are used in the figure, and full length blots for the same are presented in S. Figure 6. Individual data points (black color dots) are indicated on the bar graph representing mean ± SD.*p < 0.05; Student’s t-test, n = 3.

In the present study, the mechanism in which increase in outflow facility upon treatment with SB77 in anterior segments received cyclic IOP was investigated. Anterior segments showed activation of RhoA in response to cyclic IOP and the presence of SB77 significantly inactivated such response (*p* = 0.044). This further indicates that, the increase in outflow resistance in response to cyclic IOP is mediated through the activation of RhoA/ROCK signaling and such activation was inhibited by SB77 treatment (Fig. 4).

### Effect of Cyclic IOP on ECM proteins in presence or absence of SB77

**Figure 5:**
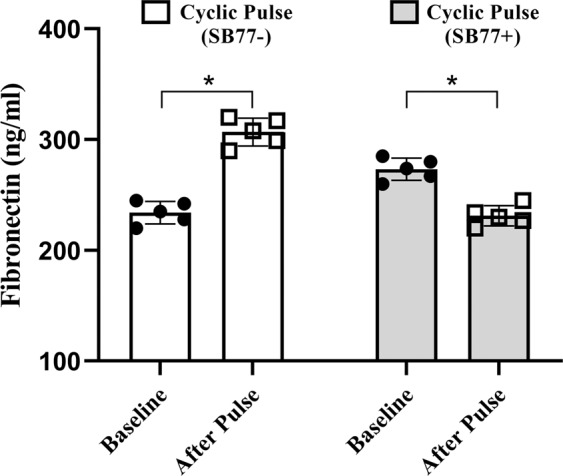
Estimation of Fibronectin Levels in Conditioned Media. ELISA analysis revealed that the levels of fibronectin was elevated in response to cyclic IOP as compared to its control eyes (p = 0.015). The presence of SB77 significantly reduced the fibronectin levels (*p* = 0.049). Individual data points (black color dots) are indicated on the bar graph representing mean ± SD; n = 5 pairs *P < 0.05; Paired-t-test.

In this study, the levels of secreted fibronectin in the conditioned media collected at baseline and after pulse in presence or absence of SB77 was investigated using Sandwich ELISA. Anterior segment received cyclic pulse showed a significant increase (*p* = 0.015) in secreted fibronectin level (308 ± 9 ng/ml) from its baseline (235 ± 7 ng/ml) in the absence of SB77. A significant decrease (*p* = 0.049) in fibronectin level from baseline (275 ± 7 ng/ml) was observed in SB77 (231 ± 10 ng/ml) treated anterior segments which indicates that the increase in outflow facility is associated with the reduction in the secretion of fibronectin upon SB77 treatment (Fig. 5).

**Figure 6:**
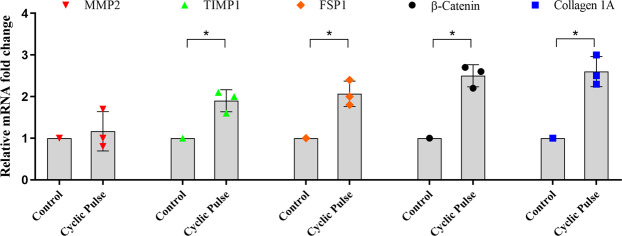
Effect of Cyclic IOP on mRNA expression of markers associated with fibrosis. The mRNA expression profile of fibrotic markers in pulsed and control anterior segments was carried out by qPCR. All fibrotic markers were significantly up-regulated in pulsed anterior segments as compared to its experimental control. The expression of MMP was unaffected by the cyclic pulsations. mRNA levels were normalized to GAPDH. Individual data points (color dots) are indicated on the bar graph representing mean ± SD. *p < 0.05; Student’s t-test; n = 3.

The presence of cyclic IOP induced a significant increase in the mRNA expression of FSP-1 (*p* = 0.012), COL1A1 (*p* = 0.013) and β-catenin (*p* = 0.015) as compared to control eyes with steady state perfusion. The levels of MMP2 did not show any difference between experimental and control eyes. However, the levels of TIMP1 was increased significantly (*p* = 0.029) (Fig. 6).

**Figure 7:**
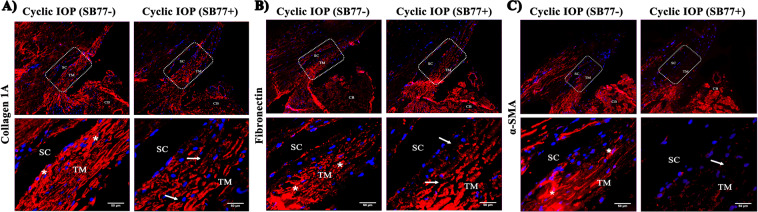
Effect of SB77 on the expression of Collagen 1A, fibronectin and α-SMA by Immunofluorescence Analysis. Representative immunofluorescence images taken at lower and higher magnifications for (**A**) Collagen 1A (**B**) fibronectin and (**C**) α-SMA are shown. White box indicates the magnified region covering TM and SC for each marker. Increased Collagen 1A, fibronectin and α-SMA expression in trabecular meshwork beams and also in the inner wall of Schlemm’s canal endothelium was observed in tissues that received cyclic pulse (*). There was also a reduction in the inter trabecular spaces. The presence of SB77 significantly reduced the positivity of all markers studied with increased inter- trabecular spaces (white arrow). SC = Schlemm canal; TM = Trabecular meshwork and CB = Ciliary body. Scale bar = 50 μm.

Immunofluorescence analysis showed increased fluorescent intensity of COL1A1, fibronectin and α-SMA in aqueous outflow pathway tissue after cyclic IOP as compared to control. The presence of SB77 blunted the expression of these markers indicating that the increase in outflow resistance was due to the increased expression of ECM (Fig. 7).

## Discussion

Although ROCK inhibitors are in clinical practice for the management of glaucoma, their efficacy in modulating cyclic mechanical stress condition is not well studied. Therefore, the primary goal of the present study was to investigate the efficacy of a new rho kinase inhibitor, SB77 in modulating the aqueous outflow resistance mediated by the cyclic mechanical stress using HOCAS.

In our previous study, we have demonstrated the IOP lowering property of SB77 in perfused human anterior segments under normal conditions. SB77 was effective in lowering the IOP in a dose-dependent manner. The enhanced outflow facility was partly mediated through the ROCK/MLC II pathway^20^. Therefore, in the present study the efficacy of SB77 in alleviating the aqueous outflow resistance caused by the cyclic mechanical stress in perfused human eyes was assessed.

The blood pulsations with each heart beat is reported to transmit waves that create transient, repetitive changes in IOP at the rate of approximately 2.7 mm Hg/s^8^. The difference between systolic and diastolic IOP is defined as the ocular pulse amplitude (OPA) and the values of OPA in healthy subjects range from 0.9 to 7.2 mm Hg^21^. Therefore, in the present study we have successfully introduced the cyclic IOP pulsations as a physiologically relevant pulsatile stress in human anterior segment perfusion culture with the help of blood pulsatile pump *ex vivo*. As expected, the cyclic pulsations for 8 h caused a significant reduction in TM outflow facility (−19.79 ± 4.93%; p = 0.0190) as compared to its experimental control. The percentage reduction in TM outflow facility was less as compared to the previous study (30%)^8^. This could be due to the difference in the magnitude of the ocular pulse used to induce pulsatile stress in perfused human anterior segment. The previous study utilized the magnitude of 2.7 mm Hg for 8-10 h. However in the present study, the magnitude of 4 mm Hg for 8 h was used. Both magnitudes of ocular pulse fall within the normal range and comparable. However, the systematic studies in which the reduction in outflow facility with incremental pressure is warranted to explore the extent of TM sensitivity towards cyclic mechanical stress.

In order to investigate whether the outflow resistance upon cyclic mechanical stress is mediated through RhoA/ROCK signaling in TM, we analysed the status of RhoA and its downstream effector molecule p-MLC. We found that the experimentally induced cyclic mechanical stress significantly activated RhoA and its effector molecules p-MLC (Fig. 3). MLC phosphorylation status is an indicator of cellular contraction, formation of actin stress fibres and focal adhesions^22,23^. Rho kinase mediates smooth muscle contraction by phosphorylating the myosin light chain directly and by decreasing myosin phosphatase activity indirectly, resulting in increased actin stress fibre formation^24^. This is in agreement with the previous findings that during mechanical stress, the cell-matrix adhesion complexes sense and transmit the mechanical stimulation to the cytoskeleton. As a consequence of these events activates RhoA from the GDP to the GTP-bound state and induces downstream molecular changes^25^. The altered activity of RhoA/ROCK in cyclical stretching microenvironment was documented in other cell types such as endothelial cells, smooth muscle cells, and cardiomyocytes also^26,27^. The presence of SB77 treatment significantly reduced RhoA (Fig. 4). Our findings suggest that the increase in outflow resistance was due to at least in part through the activation of RhoA/ROCK signaling and inhibition of RhoA by SB77 rescued this response and enhanced outflow facility. Thus SB77 treatment is effective enhancing the outflow facility under normal conditions but also in relieving the cyclic IOP mediated mechanical stress condition. However, the triggering mechanism by which the cyclic mechanical stress activates RhoA is not known. Future studies to investigate the role of cytokines especially TGF-β2 in activating RhoA/ROCK signaling would help in advancing the knowledge in this line.

RhoA activation under mechanical stress is also associated with increased cell proliferation which is reported to be mediated through several signalling pathways. A link between RhoA activation, mechanical signaling and cell proliferation have been documented in cancer cells as well as in other cell types^28^. This is in agreement with the observed increased TM cellularity by previous investigators in response to cyclic IOP and also by us in the present study (data not shown).

Mechanical stimuli can influence the expression of specific ECM proteins, matrix metalloproteinases (MMPs) and other proteins involved in ECM turnover^29^. Fibronectin is one of the major ECM proteins found in the TM and its expression is found to be up-regulated during aging and in some patients with POAG^30^. The secreted levels of fibronectin upon cyclic IOP pulsations were investigated in the present study. Significantly elevated levels of fibronectin was detected in the conditioned media after cyclic pulse which clearly indicate that the modulation of ECM turnover in response to cyclic mechanical stress. When SB77 was infused in addition to cyclic IOP, there was a significant reduction in the levels of fibronectin (p = 0.049) (Fig. 5). This clearly indicates that the elevated fibronectin in response to cyclic IOP is mediated at least in part through RhoA/ROCK signaling and hence the presence of SB77 blunted such elevation in the TM. Interestingly, we found no alteration in the mRNA expression of ECM proteins and RhoA activation in the neighbouring tissue as ciliary body (Figs. S3 and S4). The experimentally induced cyclic IOP not only elevated the levels of fibronectin but also induce changes in the expression of fibrosis marker such as FSP1 and Col1A which indicates the cyclic mediated fibrotic responses in the TM (Fig. 6). In our previous studies, we have clearly demonstrated that SB77 is effective in reducing the fibrotic markers as compared to the standard experimental rho kinase inhibitor Y27632^20^ and the present study also confirmed these findings.

As a consequence of homeostatic response of IOP, there is a MMPs-initiated turnover of ECM, changes in cytoskeleton and cytokine secretion in the TM^31,32^. The elevated levels in the expression of TIMP-2 in the present study indicate the modulation of ECM turnover in cyclic mechanical stress environment but the expression of MMP-2 was unaltered. Other MMPs are known to play a crucial role in regulating outflow resistance. Detailed studies to investigate the role of other MMPs associated with mechanical stress especially MMP-1, MMP-3 and MMP-9 are warranted.

β-catenin is the key transcription factor for Wnt/β-catenin signaling in trabecular meshwork and its dysregulation is associated in the pathogenesis of glaucoma^33^. The increased expression of β-catenin in the present study indicates the possible involvement of Wnt/β-catenin signaling in cyclic medicated stress. It is reported that there is a cross-talk between Wnt/β-catenin signaling and TGFβ/Smad pathways. Cyclic mechanical stress in the TM also induce TGF-β2 and IL-6 in both primary cultures of human TM cells and perfused human anterior segments^34,35^. Further studies are warranted to identify the role of cytokines in regulating ECM in TM in maintaining the homeostasis of IOP.

In conclusion, physiologically relevant pulsatile stress was successfully induced in perfused human cadaveric eyes and resulted in a reduced outflow facility. Treatment with the rho kinase inhibitor SB77 significantly reduced the outflow resistance medicated by cyclic mechanical stress with the inactivation of RhoA/ROCK signaling and decreased expression of ECM and fibrotic markers. This study indicates that SB77 is efficacious by enhancing the outflow facility in both normal and mechanical stress condition and thus may be a potential clinical candidate for the management of glaucoma.

## Methods

### Ethical statement

All donor eyes used in the study were consented for research and education if found unsuitable for transplantation due to insufficient corneal endothelial cell count. The written consent of the donor or next of kin was also obtained. The study was conducted in accordance with the Declaration of Helsinki, and the protocol was approved by the Institutional Review Board of the Aravind Medical Research Foundation, Madurai (ID NO. RES2014054BAS).

### Human donor eyes

Post-mortem human eyes were obtained from the Rotary Aravind International Eye Bank, Aravind Eye Hospital, Madurai. Donor eyes were enucleated within 5.3 h of death (mean elapsed time between death and enucleation was 4.3 ± 1.0 h) and kept at 4 °C until culture. Paired donor eyes were used for all human organ-cultured anterior segment (HOCAS) experiments. All eyes were examined under the dissecting microscope for any gross ocular pathological changes and eyes without such changes were used for the experiments. The donor eyes which failed to stabilize within 36 h of culture were excluded from the study. The characteristics of donor eyes used for the study are given in Table S1.

### HOCAS setup with pulsatile perfusions

HOCAS was established using paired human donor eyes as described previously^20^. The effect of cyclic biomechanical stress was studied in post-mortem paired human eyes using a modified HOCAS model as described previously^8^. A positive displacement piston pump (Pulsatile Blood Pump, Harvard Apparatus; USA) was used in addition to syringe pump to deliver IOP pulsations (Fig. S1). Ocular pulsations were introduced with a peak-to-peak magnitude of 4 mm Hg at a frequency of 1 Hz. One eye of each pair received pressure oscillations for 8 h, while the contralateral eye received a steady perfusion (flow rate: 3 μl/minute). The eye pressure was recorded every 10 minutes throughout the experiment using pressure transducers (APT300, Harvard Apparatus, MA, USA) connected to the power lab system (AD Instruments, Co., USA) with LabChart Pro software ver.6. At the conclusion of the cyclic pulse, the anterior segments were resumed to steady-state perfusion for another 24 h to measure the outflow facility. Then the anterior segments were snap-frozen in liquid nitrogen and tissues stored at −80 °C until further use. In all experimental eyes, one half of the tissue was preserved for protein and the other half was preserved for RNA extraction.

### Drug treatment

**Figure 8:**
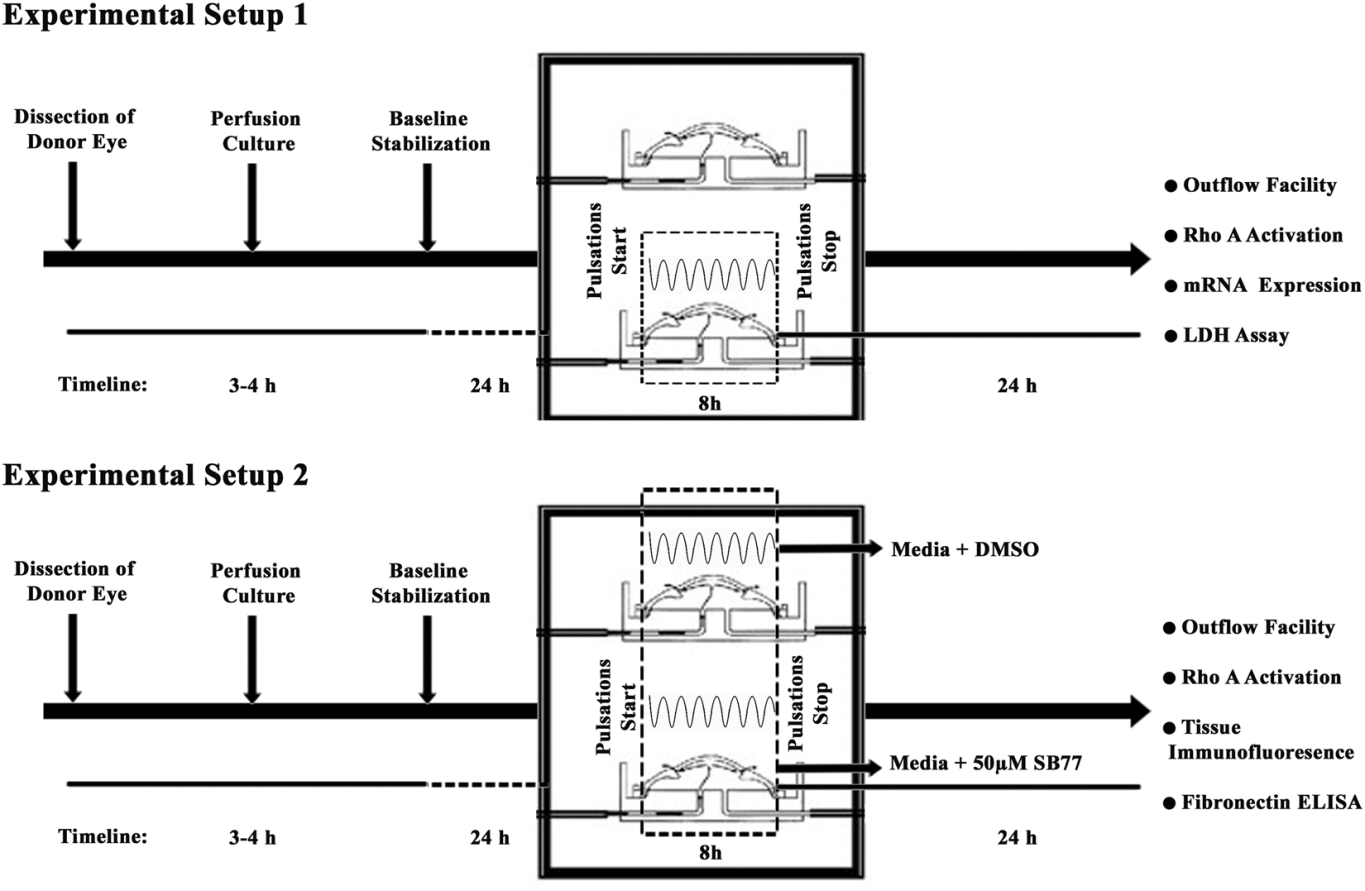
Schematic Representation of Experimental Plans used for the Study. Experimental Plan showing cyclic pulsations treatment (Experimental Plan 1) and cyclic pulsations with SB77 treatment (Experimental Plan 2).

In another set of experiments, the effect of a new Rho kinase inhibitor, SB77 in overcoming the cyclic pulsations (stress) mediated response was studied. The postmortem paired human eyes were perfused at a constant inflow rate (3 μl/minute) until they reached a stable baseline pressure. Then, both anterior segments were introduced with cyclic pulsations for 8 h as described previously^8^. After that, one eye of each pair was treated with medium containing 50 µM SB77 and the other eye received medium containing vehicle as control. The outflow was monitored 24 h post treatment. The schematic experimental plan is given in Fig. 8. At the end of the experiment, one half of the anterior segment was fixed overnight with 4% PFA and the other half was snap-frozen and stored at −80 °C until further analysis.

### Measurement of aqueous outflow facility

Aqueous outflow facility (µl/minute/mm Hg) was calculated as the ratio between the inflow rate (µl/minute) and the measured IOP (mm Hg). Outflow was calculated every hour as the average of 6 values recorded every 10 minutes, beginning 3 h before the cyclic pulsations or drug treatment as baseline. The effect of cyclic pulsations on outflow was calculated for 24 h when the anterior segments were on steady-state perfusions immediately following 8 h of cyclic pulse and compared with the respective control eyes under steady state perfusion. The effect of cyclic pulsations and the effect of SB77 on outflow was calculated after 24 h and expressed as the percentage change from corresponding baselines.

### Tissue viability by LDH assay

The effect of cyclic IOP on tissue viability was assessed by the LDH assay using commercially available kit as per the manufacturer’s instructions (Thermo Fisher Scientific Inc, IL, USA). The effluent media from perfused anterior segments was monitored for LDH release before and after pulsations. Collected perfusate was centrifuged at 500 g for 5 minutes and stored at −80 °C until LDH assay. Briefly, the samples were incubated with tetrazolium salt (INT) to obtain a colored formazan product due to the activity of LDH present in the samples, which was then quantified on a microplate reader at 490 nm (Spectramax M3, Molecular devices, PA, USA). The conditioned media collected from HOCAS perfused with DMEM medium containing 1% Triton X-100 for 3 days was used as a positive control and perfusion media used as negative control for the assay.

### Immunohistochemistry

The standard tissue immunohistochemistry protocol was followed as described previously^20^. Briefly, 5-μm tissue sections were de-paraffinized and rehydrated using three changes of xylene followed by three changes of graded series of ethyl alcohol respectively. To unmask the antigen epitopes, heat induced antigen retrieval was performed with 0.1 M citrate buffer pH 6.0 for 20 minutes at 100 °C. Tissue endogenous biotin was blocked using avidin –biotin blocking system for 10 minutes. Tissue sections were incubated with primary antibody diluted in 2% BSA in TBS for anti-collagen 1 A (Abcam, Cambridgeshire, UK) (1:500) or anti-fibronectin (Santa Cruz Biotechnology, TX, USA) (1:200) or anti-α Smooth Muscle Actin (SMA) antibody (R&D Systems, MN, USA) (1:200) overnight at 4 °C in a humidified chamber. Tissues were washed thrice with PBS and incubated with Dylight 594-conjugated secondary antibody (Vecta Laboratories, Inc., CA, USA) (1:500) for 60 minutes. After washing, the tissues were mounted with anti-fade mounting media containing DAPI and observed under fluorescence microscope (AXIO Scope A1, Zeiss, Germany). Tissue without primary antibody served as a negative control.

### Activation of RhoA by Pull-down assay

The effect of cyclic IOP and SB77 on aqueous outflow was assessed by the activation of RhoA in tissue lysates of TM after respective treatments as described previously^20^. RhoA activation status was evaluated by a pull-down assay using the RhoA Activation Assay kit (B124, Cytoskeleton, CO, USA) according to the manufacturer’s protocol. The TM tissue lysates were prepared by pooling the pre-stored halves of the anterior segments (6-Control, 6-Pulsed) after HOCAS with respective treatments. Briefly, the total protein from each of the treatments were incubated with Rhotekin-binding domain (RBD) beads to pull down active RhoA (Rho-GTP form), followed by immunoblot analysis using an anti- RhoA monoclonal antibody (Cytoskeleton Inc, CO, USA). Total RhoA present in the lysates was taken as loading control, detected using anti-RhoA polyclonal antibody (sc179; Santa Cruz Biotechnology, TX, USA) and the data was normalized to total RhoA.

The phosphorylation status of myosin light chain (p-MLC) which is the downstream effector molecule of RhoA was also investigated. Samples with equal quantity of total protein were pooled in Laemmli buffer and separated by 12% SDS-PAGE, followed by transfer of resolved proteins onto nitrocellulose membranes. Membranes were blocked for 1 h in Tris-buffered saline containing 0.1% Tween 20 and 5% (w/v) BSA at room temperature, and subsequently probed with rabbit anti-phospho-myosin light chain or myosin light chain antibody for overnight at 4 °C (1∶500 dilution; Cell Signaling Technology, MA, USA). Membrane were washed and probed with horseradish peroxidase-conjugated secondary antibodies. Detection of immunoreactivity was performed by enhanced chemi-luminescence western blotting substrate (Pierce; Thermo Fisher Scientific, IL, USA). Densitometry was performed using NIH Image J software (http://imagei.nih.gov.ij/) provided in the public domain by NIH, Bethesda, MD, USA). Data were normalized to the total MLC.

### Expression Profiling of Markers associated with Fibrosis by Quantitative RT-PCR

Expression levels for fibroblast specific protein 1(FSP1), collagen 1A1 (COL1A1), β-Catenin, matrix metalloproteinase 2 (MMP2) and tissue inhibitor of metalloproteinase 1(TIMP1) in isolated TM tissue from the anterior segments received cyclic pulsation and steady state perfusion were analysed by quantitative PCR (qPCR). Briefly, the TM tissues obtained from the respective experiments were homogenized with trizol reagent and loaded onto a QIAshredder column (Qiagen, Inc., CA, USA). Then total RNA was extracted using the RNeasy Mini Kit (Qiagen, Inc., CA, USA) was quantitated using NanoDrop 2000 UV-Vis Spectrophotometer (Thermo Scientific, Wilmington, DE). Equal amounts of RNA were then reverse transcribed using SuperScript III First-Strand Synthesis System (Invitrogen, CA, USA) and Quantitative PCR was performed using QuantiTect SYBR Green PCR Kit (Qiagen, Inc., CA, USA) using 2 μl of cDNA and 0.5 μM each of the forward and backward primer. The primer sequences for each gene is listed in Table 2. PCR reactions were done in triplicate using the following protocol: 95 °C for 7 minutes followed by 45 cycles of 95 °C for 15 seconds and 60 °C for 30 seconds. The specificity of primers was validated by a dissociation curve analysis and the fold change in expression of each gene was calculated using the 2^−ΔΔCT^ method, with GAPDH as an internal control.

**Table 2:** List of Primers used for the Gene Expression Studies.

Gene Name	Gene Accession number	Forward primer	Reverse Primer	Product size (bps)
FSP1	NM_019554.3	GAGAAGGCCCTGGATGTGAT	CCTCGTTGTCCCTGTTGCTG	192
Col1A1	NM_000088.4	GAGAGCATGACCGATGGATT	CCTTCTTGAGGTTGCCAGTC	185
β-Catenin	NM_001330729.2	CATGGAACCAGACAGAAAAGC	GCTACTTGTTCTTGAGTGAAG	200
TIMP 1	NM_003254.3	GCTTGGAACCCTTTATACATCTTG	CCTTCTGCAATTCCGACCT	120
MMP2	NM_004530.6	CCAAGGTCAATGTCAGGAGAG	GCACCCATTTACACCTACAC	100
GAPDH (Control)	NM_001357943.2	TGCACCACCAACTGCTTAGC	GGCATGGACTGTGGTCATGAG	90

### Fibronectin ELISA

Effluent media collected from anterior segments perfusion culture with cyclic pulse in presence or absence of SB77 were centrifuged at 500 g for 5 minutes to remove debris and stored at −80 °C until analysis. On the day of analysis, the samples were brought to room temperature and assayed for the amount of secreted fibronectin using commercially available ELISA kit (R&D System MN, USA) as per the manufacture’s protocol after estimating the total protein concentration by BCA method. The estimated fibronectin levels were normalized to total protein concentration.

### Statistical analysis

Statistical analysis was carried out using Graph Pad Prism version 8.0.1 (GraphPad Software, San Diego, CA, USA). Data are presented as mean ± SD values unless otherwise specified. For outflow studies and Fibronectin ELISA, a paired-t-test was used to calculate the significance of difference from the baseline values in both experimental and control eyes. For immunoblot, LDH Assay and qPCR, the significance in fold change between treated and controls were calculated by Student’s-t-test. *p* < 0.05 or less was considered statistically significant.

## Supplementary information

Supplementary Information.
