# Supplementary material for: SB772077B (SB77) Alleviated the Aqueous Outflow Resistance Mediated by Cyclic Mechanical Stress in Perfused Human Cadaveric Eyes
Source: Sci Rep. 2020 Jun 23;10:10202. doi: 10.1038/s41598-020-67087-2 (PMC7311383; doi:10.1038/s41598-020-67087-2)
Supplement: Supplementary file 1 — Supplementary Information. [file 41598_2020_67087_MOESM1_ESM.pdf]

Modified version

Supplementary Information

SB772077B (SB77) Alleviated the Aqueous Outflow Resistance Mediated by Cyclic Mechanical Stress in Perfused Human Cadaveric Eyes

Soundararajan Ashwinbalaji[1], Ravinarayanan Haribalaganesh[1], Subbaiah Krishnadas[2], Veerappan Muthukkaruppan[3], Srinivasan Senthilkumari[1] *

[1]Department of Ocular Pharmacology, Aravind Medical Research Foundation, #1, Anna Nagar, Madurai-20, Tamilnadu, INDIA. E-mail: ss_kumari@aravind.org; aashwinbtech@gmail.com; haribalaganesh@gmail.com

[2]Glaucoma Clinic, Aravind Eye Hospital, #1, Anna Nagar, Madurai-20, Tamilnadu, INDIA. E-mail: krishnadas@aravind.org

[3]Advisor, Aravind Medical Research Foundation, #1, Anna Nagar, Madurai-20, Tamilnadu, INDIA. E-mail: muthu@aravind.org

* **Corresponding Author:**

Srinivasan Senthilkumari, M. Pharm, Ph. D

Department of Ocular Pharmacology, Aravind Medical Research Foundation

#1, Anna Nagar, Madurai-625020, Tamilnadu, India

Tele (0): +91-452-4356550; extn. 438; Fax: +91-452-2530984; e.mail: ss_kumari@aravind.org

Supplementary Figures

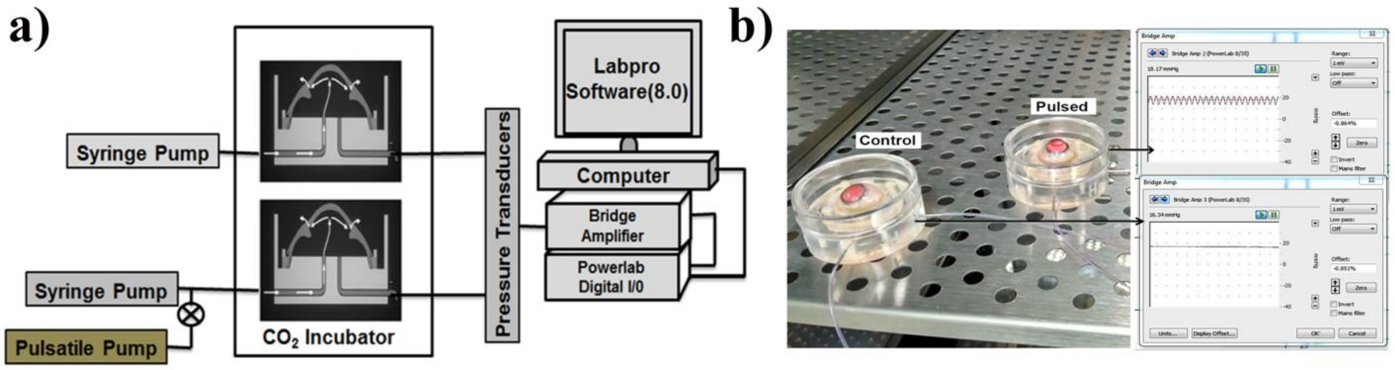

Figure S1. Schematic Representation of (a) Equipment Setup used for the study and (b) Perfusion Cultured Human anterior Segment with or without cyclic pulsations. The tracings were recorded overtime on a computer connected to the Power Lab system (AD Instruments, Co, USA) with Lab Chart Pro Software (ver.6).

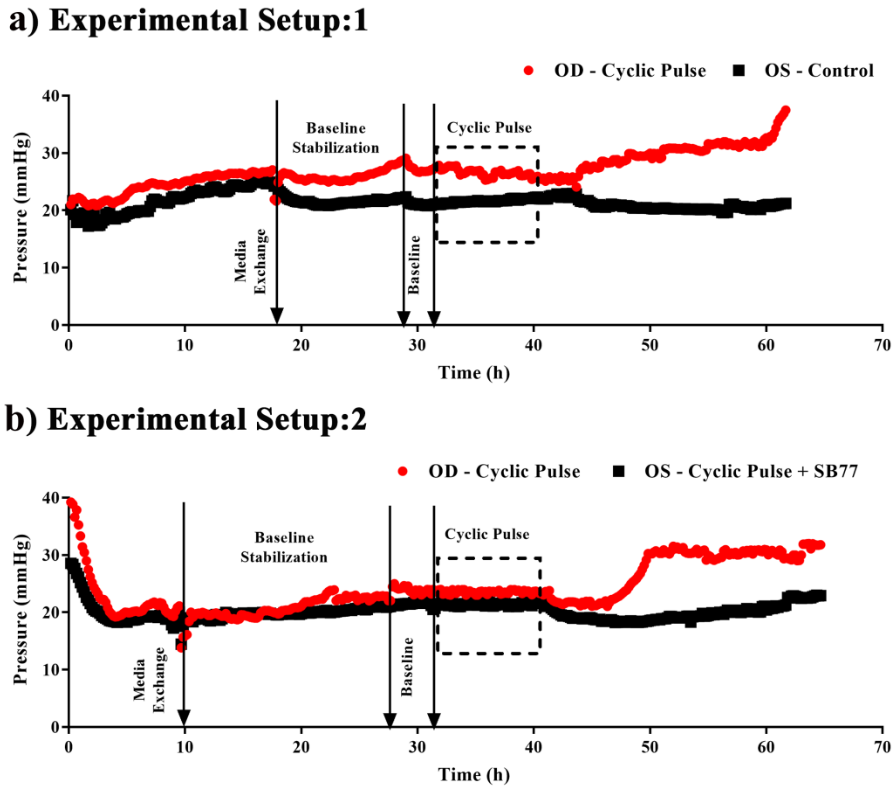

Figure S2. Representative IOP graph for both experimental conditions.

(a) Anterior segment received cyclic pulsations showing a gradual increase in IOP from baseline.

(b) SB77 treatment attenuated the IOP increase in anterior segments received cyclic pulsations compared to untreated anterior segments. Dashed lines indicate the beginning and end of the pulsations.

a)

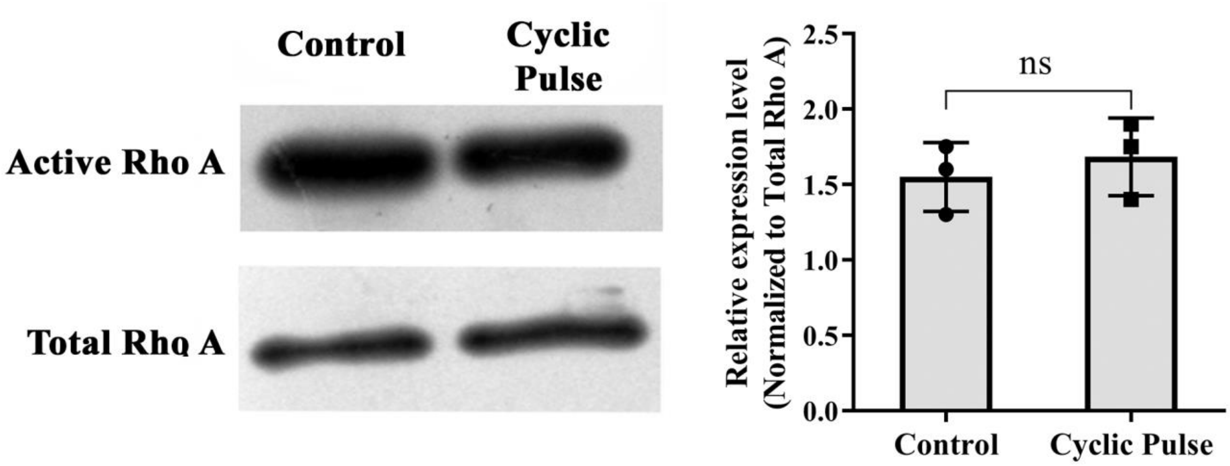

b)

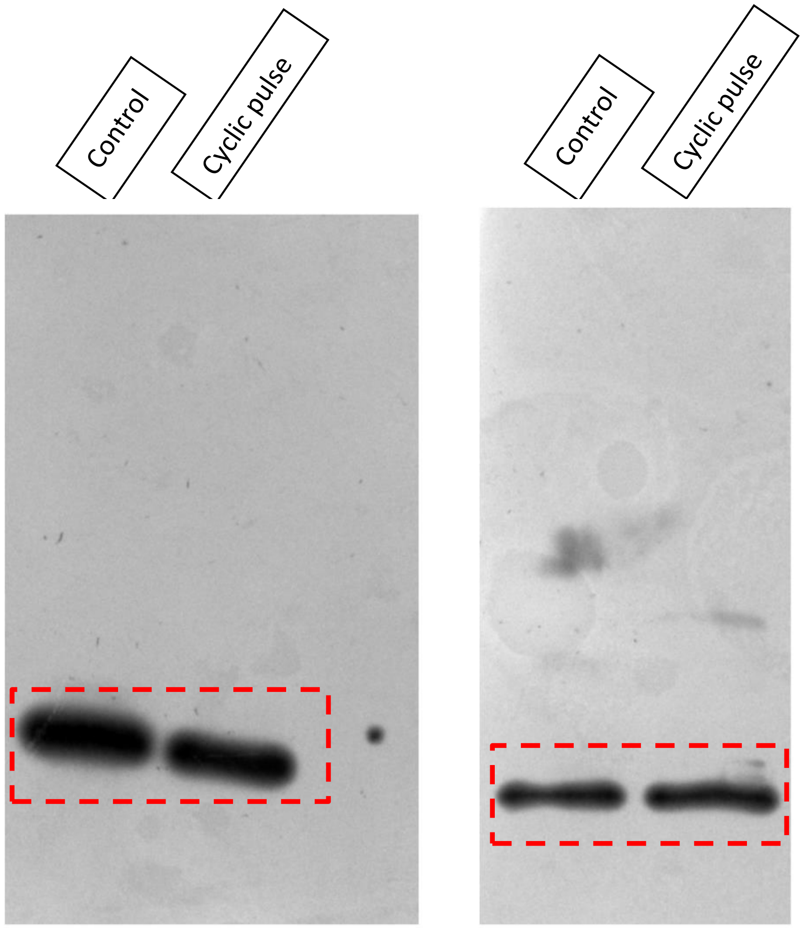

Figure S3. Effect of cyclic IOP the status of RhoA in ciliary body.

(a)The levels of activated RhoA was unaltered in ciliary body in response to cyclic IOP as compared to eye's at steady state perfusion. Levels of activated RhoA were normalized to total

Rho A. Individual data points (black dots) are indicated on the bar graph representing mean ± SD. *$p<0.05$; Student's t-test; n=3. ns, not significant. (b) Full length blot of Fig S3 A. Red-dotted lines indicate the cropped location of the blot.

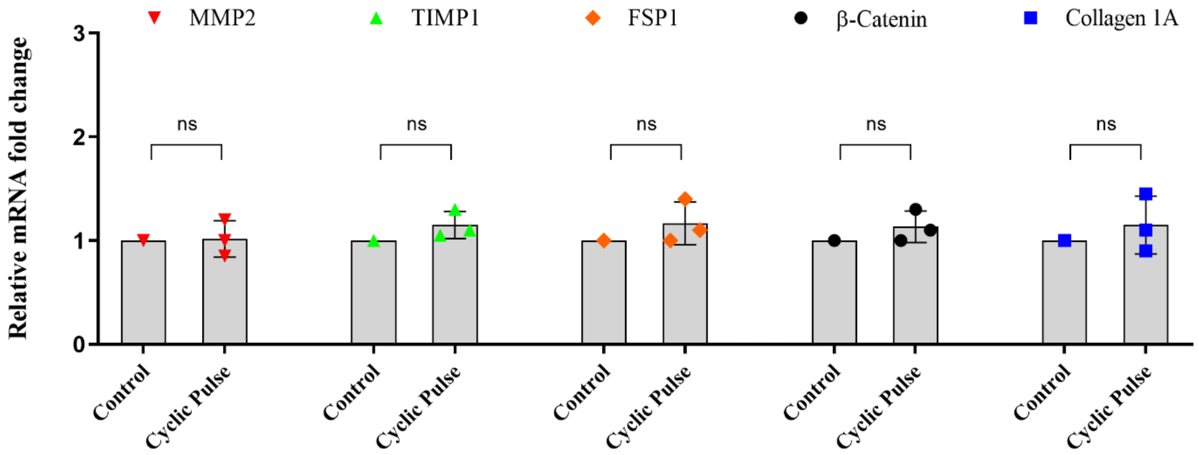

Figure S4. Effect of Cyclic IOP on mRNA expression of markers associated with fibrosis in ciliary body.

mRNA expression profile of fibrotic markers from pulsed and control ciliary body was carried out by qPCR. No change in the expression of fibrotic markers. mRNA levels were normalized to GAPDH. Individual data points (colour dots) are indicated on the bar graph representing mean ± SD. Student's t-test; n=3, ns= not significant.

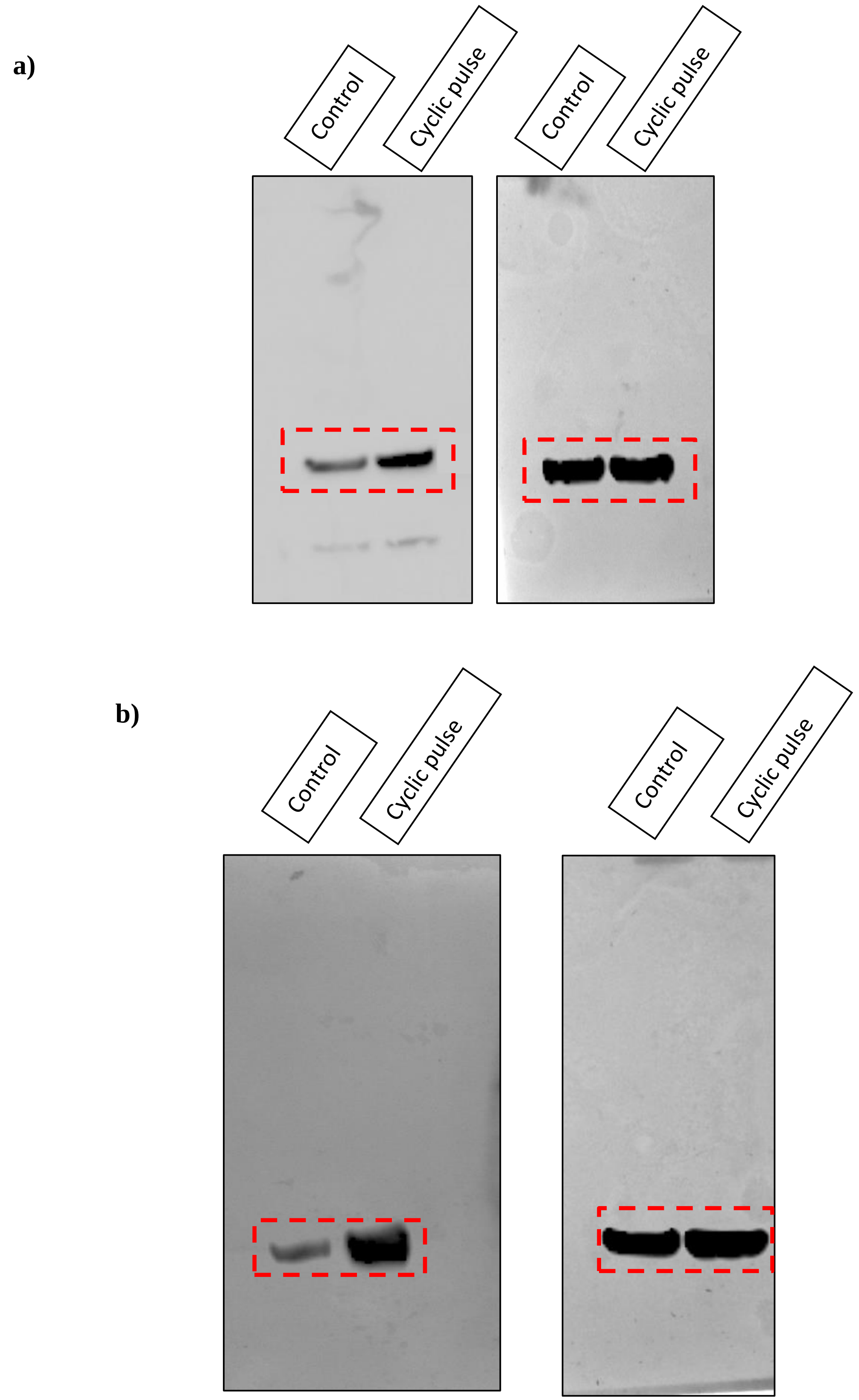

Figure S5. Full length blots of Fig.3: Western blot analysis of (a) active RhoA~~,~~ and total RhoA ~~and~~ (b) p-MLC and total MLC. Red-dotted lines indicate the cropped location of the blot.

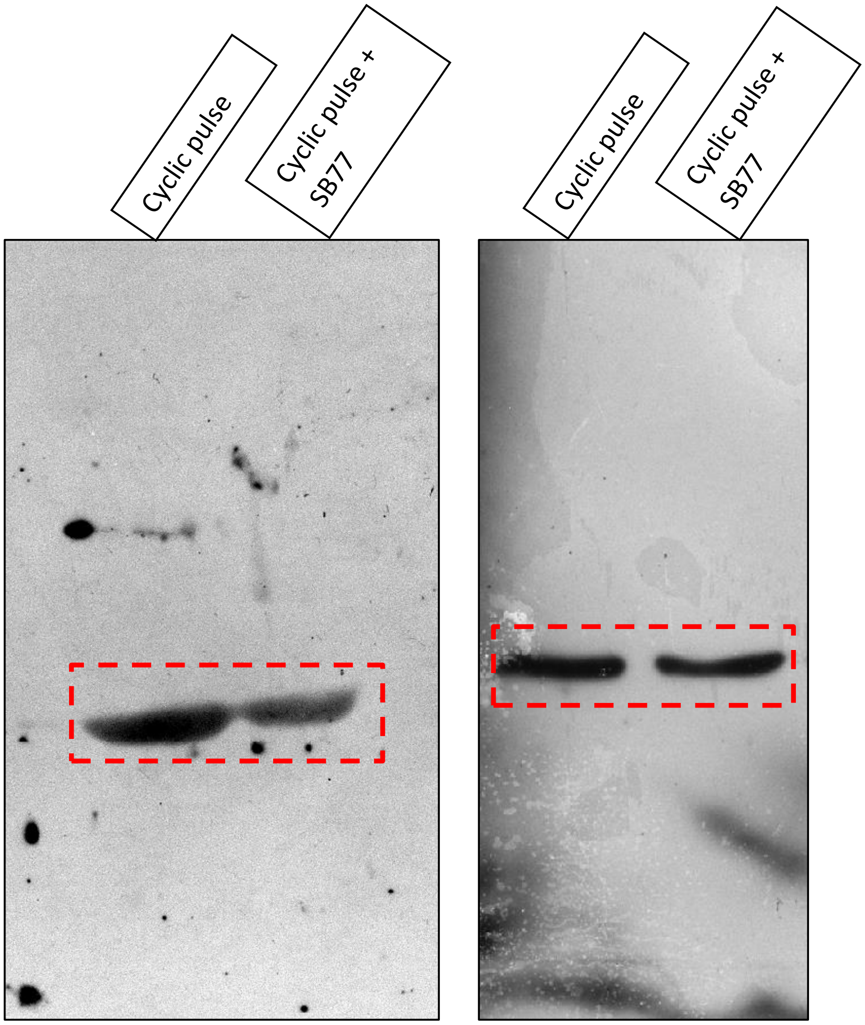

Figure S6. Full length blots of Fig.4: Effect of SB77 on the status of RhoA activation. Western blot analysis of active RhoA and total RhoA in response to IOP pulsations with or without SB77 treatment. Red-dotted lines indicate the cropped location of the blot.

Supplementary Table

Table S1. Characteristics of Human Donor Eyes Used for the Study

**SI.No**	**Age**	**Sex**	**COD**	**TOD-TOE (h)**	**TOD-TOC (h)**
HOCAS with Cyclic IOP Experiments					
1	75	M	Cardiac Arrest	3.5	29
2	40	M	Cerebral Injury	4	22
3	60	M	Cardiac Arrest	4	30
4	85	F	Respiratory Arrest	3	25
5	72	M	Cardiac Arrest	6	46
6	53	F	Cancer	5	27
HOCAS with Cyclic IOP + SB77 Experiments					
7	72	M	Cardiac Arrest	5	27
8	85	M	Cerebral Injury	3.5	24
9	70	M	Cardiac Arrest	4.5	28
10	85	F	Respiratory Arrest	3	25
11	81	M	Cardiac Arrest	6	46

COD-Cause of Death; **TOD**-Time of Death; **TOE**-Time to enucleation and **TOC**-Time to culture.

The mean (±SD) age of the donors used for the study was 70.7±14.5 years. The eyes were enucleated within 5.3 h (4.3±1.0). The mean elapsed time between enucleation and culture was 26.3±2.5 h except 2 (Sl.no 5 and 11).
